# Receipt of Training Among Family Caregivers Assisting With a Post-Hospital Care Transition

**DOI:** 10.1093/geroni/igab046.348

**Published:** 2021-12-17

**Authors:** Julia Burgdorf, Chanee Fabius, Catherine Riffin, Jennifer Wolff

**Affiliations:** 1 Center for Home Care Policy & Research, New York, New York, United States; 2 Johns Hopkins Bloomberg School of Public Health, Baltimore, Maryland, United States; 3 Weill Cornell Medicine, New York, New York, United States

## Abstract

Medicare Conditions of Participation require hospitals to provide training to family and unpaid caregivers when their support is necessary to enact the post-discharge care plan. However, caregivers often report feeling unprepared for this role. We perform a cross-sectional analysis of the 2017 National Health and Aging Trends Study and its linked National Study of Caregiving (nationally representative surveys of older adults and their family and unpaid caregivers, respectively) to assess the prevalence of, and factors associated with, caregiver receipt of adequate transitional care training. Our analytic sample includes 795 (weighted n=7,083,222) family caregivers who assisted an older adult during a post-hospital care transition in the past year. The outcome of interest caregiver-reported receipt of the training needed to manage this transition (“adequate transitional care training”) from hospital staff. Six in ten (59.1%) caregivers who assisted during a post-hospital care transition reported receiving adequate transitional care training. In weighted, multivariable logistic regression models, caregivers were half as likely to report receiving adequate transitional care training if they were black compared to white (Adjusted Odds Ratio (aOR): 0.52; 95% CI: 0.31-0.89) or experienced financial difficulty (aOR: 0.50; 95% CI: 0.31-0.81). Findings suggest that socially vulnerable family caregivers of older adults are less likely to report receiving adequate transitional care training. Changes to the discharge process, such as using standardized caregiver assessments, may be necessary to ensure equitable support of family caregivers.

